# In Vitro Production of Calcified Bone Matrix onto Wool Keratin Scaffolds via Osteogenic Factors and Electromagnetic Stimulus

**DOI:** 10.3390/ma13143052

**Published:** 2020-07-08

**Authors:** Nora Bloise, Alessia Patrucco, Giovanna Bruni, Giulia Montagna, Rosalinda Caringella, Lorenzo Fassina, Claudio Tonin, Livia Visai

**Affiliations:** 1 Department of Molecular Medicine (DMM), Centre for Health Technologies (CHT), UdR INSTM, University of Pavia, Viale Taramelli, 3/B-27100 Pavia, Italy; giulia.montagna04@universitadipavia.it; 2 Department of Occupational Medicine, Toxicology and Environmental Risks, Istituti Clinici Scientifici (ICS) Maugeri, IRCCS, Via Boezio, 28-27100 Pavia, Italy; 3 Institute of Intelligent Industrial Technologies and Systems for Advanced Manufacturing (STIIMA), Italian National Research Council (CNR), Corso Pella, 16-13900 Biella, Italy; a.patrucco@stiima.cnr.it (A.P.); linda.car87@yahoo.it (R.C.); c.tonin@stiima.cnr.it (C.T.); 4 Center for Colloid and Surface Science (C.S.G.I.), Department of Chemistry, Section of Physical Chemistry, University of Pavia, Viale Taramelli, 16-27100 Pavia, Italy; giovanna.bruni@unipv.it; 5 Department of Electrical, Computer and Biomedical Engineering (DIII), Centre for Health Technologies (CHT), University of Pavia, Via Ferrata, 5-27100 Pavia, Italy; lorenzo.fassina@unipv.it

**Keywords:** pulsed electromagnetic field, osteogenic factors, wool keratin scaffolds, bone tissue engineering

## Abstract

Pulsed electromagnetic field (PEMF) has drawn attention as a potential tool to improve the ability of bone biomaterials to integrate into the surrounding tissue. We investigated the effects of PEMF (frequency, 75 Hz; magnetic induction amplitude, 2 mT; pulse duration, 1.3 ms) on human osteoblast-like cells (SAOS-2) seeded onto wool keratin scaffolds in terms of proliferation, differentiation, and production of the calcified bone extracellular matrix. The wool keratin scaffold offered a 3D porous architecture for cell guesting and nutrient diffusion, suggesting its possible use as a filler to repair bone defects. Here, the combined approach of applying a daily PEMF exposure with additional osteogenic factors stimulated the cells to increase both the deposition of bone-related proteins and calcified matrix onto the wool keratin scaffolds. Also, the presence of SAOS-2 cells, or PEMF, or osteogenic factors did not influence the compression behavior or the resilience of keratin scaffolds in wet conditions. Besides, ageing tests revealed that wool keratin scaffolds were very stable and showed a lower degradation rate compared to commercial collagen sponges. It is for these reasons that this tissue engineering strategy, which improves the osteointegration properties of the wool keratin scaffold, may have a promising application for long term support of bone formation in vivo.

## 1. Introduction

In bone tissue engineering (BTE), two fundamental properties that each biomaterial should present are biocompatibility and biodegradability. Other than these properties, most of the materials used as scaffolds for the repair of bone defects offer only a feasible passive support within which the tissue may heal or regenerate. However, an active induction and promotion of the core processes mentioned above would accelerate the tissue healing. In BTE, bioceramic scaffolds (such as calcium phosphates) are widely employed to improve bone regeneration because their chemical similarity to the bony inorganic matrix confers osteoconductive properties, increasing osseointegration.

In general, and within the context of the biodegradable natural polymers, keratin-based materials have changed the field of modern biomaterials due to their distinct properties such as biodegradability, biocompatibility, and mechanical durability. Interestingly, they can be cast as sponges, films, and hydrogels for various biomedical applications [1]. Moreover, keratin constitutes the major components of hair, wool, feathers, and nails, and can be extracted in significant amounts from animal tissues without the need for animal sacrifice [2], something which is not the case for collagen and other animal-derived osteoconductive proteins. In vivo investigation of keratose (water-soluble fraction of the keratin) was studied as a BMP2 carrier for bony regeneration of rat femoral bone defect. Results indicated There was enhanced regeneration of bone along with reduced adipose tissues [3].

This in vitro work of bone-tissue engineering starts from the wool keratin scaffold previously described [4] and aims to enrich the extracellular bone matrix (ECM) components, i.e., the over-wrap of a proteinaceous and, calcified surface, are reveal possible uses in vivo. Herein, we aimed to obtain, directly in vitro, a biomaterial summarizing the characteristics of the compact bone before in vivo implantation. As previously stated [4], wool keratin scaffolds exhibit high level of cell adhesion and proliferation; in particular, the 3D structure of this biomaterial, with controlled-size macro-porosity suitable for cell guesting and nutrient diffusion, provides a suitable environment for bone tissue engineering. In addition, and in order to accelerate and ameliorate ECM deposition we implemented the classical idea of tissue engineering using mechanical stimuli. Theoretically, the growth and development of in vitro tissue substitutes should be supported not only by biomolecules (e.g., growth factors) but also by physical factors provided by the structural context (e.g., geometric and mechanical properties of scaffolds) and by the biophysical context (e.g., the concentrated/distributed, perpendicular/tangential forces acting onto the cell plasma membrane). Fluid shear stress [5], for instance, or ultrasounds [6] or biomaterial features [7] lead to the remodeling of bone matrix in vitro.

Nonetheless, the modulation of the cell behavior on the different biomaterials is clearly proved by the osteoblasts exposed to pulsed electromagnetic field (PEMF) [8,9]. In particular and according to the “tensegrity” theory of Ingber [10], mechanical forces may induce biochemical responses targeting the transcriptional profile via mechanotransduction. The electromagnetic stimulus of this study was demonstrated [8,9] to elicit time varying mechanical forces acting perpendicularly or tangentially onto the cell membrane, in order that these forces were able to modulate the cell behavior via tensile, compressive, and shear deformations.

In this study, the electromagnetic stimulus employed to elicit time-varying mechanical forces acting perpendicularly or tangentially upon the cell membrane [8,9]. In other words, these forces were able to modulate the cell behavior via tensile, compressive and shear deformations. For instance, osteoblasts are susceptible to fluid shear stress and react with an enhanced transcription of bone matrix genes [11,12]. Both traction and compression vary the activities of intracellular signaling molecules such as Rho GTPases, guanine nucleotide exchange factors, GTPase activating proteins, and the MAPK pathway, consequently modulating the expression of transcription factors essential for the homeostasis of bone, cartilage and tooth tissues [13]. As a consequence, we can hypothesize the significance of PEMF application to improve the clinical outcome of numerous regenerative and prosthetic therapies in orthopedics and dentistry fields [14].

In sum our aim was to evaluate the feasibility of wool keratin sponges in supporting osteoblast-like cells viability and ECM deposition. Moreover, we want to verify if PEMFs, with the presence of osteogenic factors, ameliorate osteoblast-like cells responses to wool keratin biomaterial, improving their differentiation and bone matrix enrichment. Such a tissue engineering strategy could be promising for wool keratin scaffold applications in vivo, for example towards a filler for bone defects.

## 2. Materials and Methods

### 2.1. Preparation of the Keratin Sponges

Sponges (or scaffolds) were prepared following the previous procedure [4]. Botany wool, 20.3 μm mean fiber diameter, was supplied by The Woolmark Co., Milan, Italy. In brief, 8 g wool fibers snippets were bathed in 400 mL of 0.1 N NaOH, material to liquid ratio 1:50, for 24 h at 60 °C. The snippets were rinsed with tap water until pH neutral, soaked in deionized water and submitted to ultrasonic irradiation for 30 min (600 W, 20 kHz). Coarse fiber fragments were removed from the suspension by filtration with stainless steel 120 mesh sieves. The permeate was centrifuged (12,000 rpm, 15 min), and the supernatant was removed. The solid precipitate was added with deionized water and stirred until the suspension reached 0.05 g/mL. This suspension was added with 1.17 g/mL controlled-size NaCl (400–500 μm), then cast at 50 °C. The resulting material was washed with deionized water in order to remove salt. The sponge was dried at 50 °C, then at 180 °C for 2 h to improve its water stability and increase crosslinks [15].

### 2.2. Pulsed Electromagnetic Field (PEMF)

Our electromagnetic apparatus (Igea, Carpi, Italy) was used [16]. Cells were exposed to PEMF for 1 h per day at the same moment, adopting the following parameters: magnetic induction amplitude of 2 ± 0.2 mT, frequency of 75 ± 2 Hz, pulse duration of 1.3 ms (Table 1).

### 2.3. Cell Cultures

The human osteosarcoma cell line SAOS-2 was cultured in a maintenance medium (MM) constituted of McCoy’s 5A modified medium with L-glutamine (Lonza Ltd., Basel, Switzerland) and HEPES (Cambrex Bio Science, Baltimore, MD, USA), supplemented with 15% fetal bovine serum, 1% L-glutamine, 0.4% antibiotics, 2% sodium pyruvate, and 0.2% fungizone. Cells were cultured, routinely trypsinized after confluence, and maintained in an incubator at 37 °C with a 5% CO_2_ atmosphere. Before cell seeding, scaffolds (diameter, 0.8 cm; height, 1 cm) were sterilized [4] at 180 °C for 3 h, then washed twice in PBS for 10 min, placed in 48-wells, and incubated O.N. in the maintenance medium. To ensure a maximum number of attached cells for scaffolds a cell suspension of 4 × 10^5^ cells × scaffold was added in two steps onto the top of each scaffold and, after 0.5 h, 1 mL of culture medium was added to cover the scaffolds. After 24 h from seeding, the medium was changed and replaced with MM (control, ctrl) or, to induce osteogenic differentiation, with MM supplemented with osteogenic factors (OF). The osteogenic factors, dexamethasone and β-glycerophosphate were added to the maintenance medium at a concentration of 10^−8^ M and 10 mM, respectively [7]. McCoy’s 5A modified medium contains the ascorbic acid, another osteogenic supplement, at a concentration of 0.5 μg/mL. Treatment lasted up to 21 days and the medium was changed every 3 days.

### 2.4. DNA Content

Total DNA content in SAOS-2 was determined after 21 days of culture using PicoGreen assay (PicoGreen; Molecular Probes, Eugene, OR, USA). Briefly, at the end of incubation, to process material for analysis of DNA content, samples were processed through the three freeze/thaw cycles method in sterile deionized distilled water. Between each freeze/thaw cycle, scaffolds were roughly vortexed. The released DNA content was measured with the fluorometric DNA quantification kit. Samples were diluted 1:100 in 100 µL of working solution (PicoGreen reagent in TE buffer, 1:200) for the measurement. Fluorescence was detected in a dedicated 96-well plate, at 520 nm, after excitation at 480 nm, with CLARIOstar^®^ Plus Multi-mode Microplate Reader (BMG Labtech, Ortenberg, Germany). A DNA standard curve [9], obtained from a known number of osteoblasts, was used to express the results as cell number attached per scaffold.

### 2.5. Fluorescein Diacetate Assay

At day 21 of culture, live cells were visualized by a vital staining with fluorescein diacetate (FDA) on all experimental groups as described previously [17]. Briefly, 5 mg/mL FDA stock solution (Invitrogen) was prepared in acetone: 40 μL stock solution was diluted in 10 mL phosphate buffer solution (PBS) (137 mM NaCl, 2.7 mM KCl, 4.3 mM Na_2_HPO_4_, 1.4 mM NaH_2_PO_4_, pH 7.4) and 250 μL was mixed with 500 μL culture medium. Cells were incubated with a working solution for 10 min. The live cells were examined by a confocal laser scanning microscope model TSC SP5 II (Leica Microsystems, Bensheim, Germany), using a 40× oil immersion objective.

### 2.6. Scanning Electron Microscopy (SEM)

On day 21 of culture, samples were treated as previously described [16]. The samples were fixed with 2.5% (*v/v*) glutaraldehyde solution in 0.1 M Na-cacodylate buffer (pH = 7.2) for 1 h at 4 °C, washed with Na-cacodylate buffer, and then dehydrated at room temperature in an ethanol gradient series up to 100%. Scaffolds were then lyophilized 4 h for complete dehydration, and then sputter-coated with gold under high vacuum to render them electrically conductive prior to observation with Zeiss EVO-MA10 scanning electron microscope (Carl Zeiss, Oberkochen, Germany) at accelerating voltage of 20 kV for analysis of cell morphology. An energy dispersive X-ray spectroscopy (EDX) detector (X-max 50 mm^2^, Oxford Instruments, Oxford, UK) used coupled with SEM to perform the distribution maps of calcium and phosphorus onto the wool keratin scaffold surfaces. In this case (SEM-EDX analysis), the samples were examined without a conductive coating under low vacuum condition and with an accelerating voltage of 20 kV.

### 2.7. ALP Activity

ALP activity was estimated using a colorimetric endpoint assay at day 21 as previously reported [7,16]. The assay measures the conversion of the colorless substrate p-nitrophenol phosphate (pNPP) by the enzyme ALP into the yellow product p-nitrophenol (pNP). The rate of color change corresponds to the amount of enzyme present in the solution. Briefly, an aliquot (0.5 mL) of 0.3 M pNPP (dissolved in glycine buffer, pH 10.5) was added to each scaffold at 37 °C. After incubation, the reaction was stopped by the addition of 50 μL 5 M NaOH. Standards of pNPP in concentrations ranging from 0 to 50 μM were freshly prepared from dilutions of a 500 μM stock solution and incubated for 10 min with 7U of ALP (Sigma-Aldrich, St. Louis, MO, United States) previously dissolved in 500 μL of ddH_2_O. The optical densities (OD) reading was performed at 415 nm with a microplate reader (BioRad Laboratories, Hercules, California) using 100 μL of standard or samples and placed into individual wells on a 96-well plate. Samples were run in triplicate and optical densities obtained from each sample, after blank subtraction, were compared with the calibration curve of p-nitrophenol standard in order to obtain the ALP activity expressed as μM of p-nitrophenol produced per min per μg of protein.

### 2.8. Inorganic Phosphate Determination

A commercially available kit (Phosphate Colorimetric Assay Kit, Sigma Aldrich, St. Louis, MO, USA) was used to quantify inorganic phosphate levels. Briefly, the cell-seeded scaffolds were washed with TBS (Tris-buffered saline, 50 mM Tris-Cl, 150 mM NaCl, pH 7.6), and chilled on ice in 1 mL cold TBS for 15 min. TBS buffer containing Tris and NaCl does not interfere with phosphate content in the assay and then not alter the final results. Samples were then sonicated 3 times for 60 s at high setup (one cycle = 30 s sonication—10 s break—10 s sonication—10 s break). The samples were then centrifuged for 15 min at 4 °C at top speed using a cold microcentrifuge to remove any insoluble material. Supernatant was collected and transferred to a clean tube. The supernatant was diluted 1:10 in double-distilled water, and phosphates were determined. At the end of reaction, an absorbance reading was performed at 655 nm with a microplate reader (BioRad Laboratories). Samples were run in triplicate and compared against a standard-solution calibration curve. The amount of phosphate from samples was expressed as pmol/(cells × scaffold).

### 2.9. Calcium-Cresolphthalein Complexone Method

The calcium content of each sample was assayed to quantify the amount of mineralized matrix present and was measured using a Calcium Fast kit (Mercury S.p.A., Naples, Italy) according to the manufacturer’s instructions as previously reported [17]. Samples were run in triplicate and compared with the calibration curve of standards. The colorimetric end point assay measures the amount of purple-colored calcium-cresolphthalein complexone complex formed when cresolphthalein complexone binds to free calcium in an alkaline solution. Briefly, an aliquot (1 mL) of 1 N HCl was added to each sample and incubated for 24 h at RT to release calcium into solution. The sample supernatant was diluted 1/10 with the Assay Working Solution by mixing equal parts of calcium-binding reagent and calcium buffer reagent provided by the kit. Ca^2+^ standards in concentrations ranging from 0 to 10 mg/mL were prepared from dilutions of a 100 mg/mL stock solution of Ca^2+^. The absorbance reading was performed at 595 nm with a microplate reader (BioRad Laboratories) using 100 μL of standard or sample placed into individual wells of a 96-well plate. Samples were run in triplicate and compared against the standard solution calibration curve. Results are expressed as pg/cell×scaffold and presented as mean ± SD.

### 2.10. Confocal Laser Scanning Microscopy (CLSM)

For morphological observation, cell-seeded wool fibril sponges were washed for 24 h with PBS, fixed with 4% (*w/v*) paraformaldehyde solution for 30 min at 4 °C, permeabilized with 0.1% Triton X-100, then stained with Tetramethylrhodamine B isothiocyanate (TRITC) phalloidin conjugate solution (10 μg/mL, EX/EM maxima ca. 540/575, Sigma-Aldrich) in PBS for 40 min at RT, finally incubated with the primary antibody Alexa-Fluor 488 anti-β tubulin and with Hoechst 33342 for nuclei staining (2 μg/mL, Sigma-Aldrich). For osteogenic protein labeling, paraformaldehyde-fixed samples were blocked with PAT [PBS containing 1% (*w/v*) bovine serum albumin and 0.02% (*v/v*) Tween 20] for 1 h at RT. Anti-osteocalcin rabbit polyclonal antiserum (provided by Dr. Larry W. Fisher, National Institutes of Health, Bethesda, MD, USA) was used as primary antibody diluted 1:500 in PAT. The incubation with the primary antibody was made overnight at 4 °C, whereas the negative controls were incubated overnight at 4 °C with PAT instead of the primary antibodies. The scaffolds and the negative controls were washed and incubated with Alexa Fluor 488 goat anti-rabbit IgG (Molecular Probes) at a dilution of 1:750 in PAT for 1 h at room temperature. At the end of the incubation, the scaffolds were washed in PBS, counterstained for 5 min with a solution of Hoechst 33342 (2 μg/mL) to target nuclei, and then washed. The images (39 sections, images acquired every 112 μm till 4.4 mm depth) were taken using a confocal laser scanning microscope model TSC SP5 II (Leica Microsystems, Bensheim, Germany), using a 20× oil immersion objective for each sample condition, and the orthogonal projections of images were obtained using Fiji software ((Fiji Is Just) ImageJ 2.0.0-rc-69/1.52p, National Institutes of Health, Bethesda, MA, USA). The fluorescence background of the negative control was almost negligible (Figure S4). Cells seeded and cultured on Tissue Culture Plates (TCPS) after 21 days in MM and with osteogenic factors (OF) were insert as controls (Figure S4).

### 2.11. qRT-PCR

The total RNA from all samples was extracted on day 7 and 21 of culture with the NucleoSpin^®^ RNA XS kit (MACHEREY-NAGEL GmbH & Co. KG, Düren, Germany) and retro-transcribed to c-DNA with the iScript cDNA Synthesis kit (Thermo Fisher Scientific, Waltham, MA, USA). Quantitative reverse-transcription polymerase chain reaction (qRT-PCR) analysis was performed in a 96-well optical reaction plate using a qPCR Quant3 Studio (Applied BioSystem, Foster City, CA, USA). Analysis was performed in a total volume of 20 μL amplification mixture containing 2× (10 μL) Brilliant SYBR Green QPCR Master Mix (Bio-Rad Laboratories), 2 μL cDNA, 0.4 μL of each primer, and 7.2 μL H_2_O. The PCR conditions were as follows: 3 min at 95 °C, 40 cycles of 5 s at 95 °C, and 23 s at 60 °C. The reaction mixture without cDNA was used as a negative control in each run. Gene expression was analyzed in triplicate. GAPDH housekeeping was used as the housekeeping gene and results were analyzed with the 2^−ΔΔCt^ method [18] relative to the expression in the cells at day 0. The primers used are listed in Table S1.

### 2.12. Extraction of Bone Matrix Proteins and ELISA Assay

To evaluate the amount of extracellular matrix protein constituents onto the keratin scaffold in in all experimental conditions, an enzyme-linked immunosorbent assay (ELISA) was performed as previously described [7]. Briefly, samples were washed extensively with sterile PBS to remove culture medium and then incubated for 24 h at 37 °C with 1 mL of sterile sample buffer (20 mM Tris-HCl, 4 M GuHCl, 10 mM EDTA, 0.066% (*w/v*) sodium dodecyl sulphate (SDS), pH 8.0) At the end of the incubation period, the samples were centrifuged at 4000 rpm for 15 min in order to collect also the sample buffer entrapped inside the pores of the scaffolds. The total protein concentration in the collected sample buffer was evaluated with the BCA Protein Assay kit (Pierce Biotechnology, Inc., Rockford, IL, USA). Calibration curves to measure alkaline phosphatase (ALP), type-I collagen (COL-I), decorin (DCN), osteocalcin (OSC), osteonectin (OSN), and osteopontin (OSP) were prepared as previously described [7]. In order to measure the extracellular matrix amount of each protein the ELISA assay was performed as previously reported [7]. In brief, microtiter wells were coated with increasing concentrations of each purified protein, from 10 ng to 2 μg, in coating buffer (50 mM Na_2_CO_3_, pH 9.5) overnight at 4 °C. Control wells were coated with bovine serum albumin (BSA) as a negative control. To measure the ECM amount of each protein by ELISA, microtiter wells were coated, overnight at 4 °C, with 100 μL of the previously extracted ECM (20 μg/mL in coating buffer). After three washes with PBS containing 0.1% (*v/v*) Tween 20, the wells were blocked by incubating with 200 μL of PBS containing 2% (*w/v*) BSA for 2 h at 22 °C. The wells were subsequently incubated for 1.5 h at 22 °C with 100 μL with anti-phosphatase (ALP), anti-type-I collagen (COL-I), anti-decorin (DCN), anti-osteocalcin (OSC), anti-osteonectin (OSN), and anti-osteopontin (OSP) polyclonal antisera (1:500 dilution in 1% BSA), kindly provided by Dr. Larry W. Fisher. After washing, the wells were incubated for 1 h at 22 °C with 100 μL of horseradish peroxidase (HRP)-conjugated goat anti-rabbit IgG (1:1000 dilution in 1% BSA). The wells were finally incubated with 10 μL of the development solution (phosphate-citrate buffer with o-phenylenediamine dihydrochloride substrate). The color reaction was stopped with 100 μL of 0.5 M H_2_SO_4_, and the absorbance values were measured at 490 nm with a microplate reader (BioRad Laboratories). The optical densities from each sample were plotted against a calibration curve containing known amounts of each proteins. An underestimation of the absolute protein deposition is possible because the sample buffer used for matrix extraction contains SDS, which may interfere with protein absorption during the ELISA assay. The amount of extracellular matrix constituents throughout the wool keratin scaffold in the different conditions was expressed as pg/(cells×scaffold).

### 2.13. Ageing Test

Wool keratin scaffolds and commercial collagen sponges were dried at 105 °C to a constant weight then aged in isotonic fluid (0.02 mg/mL, Ringer’s solution, pH 7) at 37 °C, with the fluid replaced every 14 days [19].

### 2.14. Compression Behavior

Compression properties of the wool keratin scaffolds were determined in conditioned standard atmosphere at 20 °C, 65% RH, with an Instron 5500 R Series IX dynamometer (Instron, Pianezza, Italy). The measurements were performed on the scaffold in the absence or presence of cells in all the conditions (ctrl, PEMF, OF, and OF + PEMF) after 21 days of culture. Six samples (8 mm, diameter; 4 mm, thickness) in conditioned and wet states (bathed in distilled water for 2 h, then drained for testing) were submitted to 10 compression cycles (maximum load of 5 N) at the constant deformation rate of 10 mm/min, in order to evaluate resilience as well. Every compression cycle was stopped on reaching 3 mm stroke, starting from the top of the sponge scaffold. Samples were measured for compression force and deformation, reporting the average and standard deviation of the results.

### 2.15. Statistics

Three independent experiments (N) (unless otherwise indicated) were performed to get a statistically significant number of events, and in order to test the reproducibility of the results per each type of experiment from 2 to 3 scaffolds (n) were analyzed (as indicated in the figure legends). Results were expressed as mean ± standard deviation. Statistical analysis was carried out using GraphPad Prism 6.0 (GraphPad, Inc., San Diego, CA, USA). Analysis was performed using one-way or two-way ANOVA analysis of variance (ANOVA), followed by Bonferroni post hoc test (significance level of 0.05).

## 3. Results

To explore the effect of daily treatment with a low-frequency PEMF on human osteoblast-like cells (SAOS-2) seeded onto wool keratin scaffolds on proliferation and bone matrix deposition after 21 days of culture, four conditions were investigated: (a) cells grown in a maintenance medium (MM, control, ctrl); (b) cells grown in MM and daily exposed to PEMF; (c) cells differentiated in medium supplemented with osteogenic factors (OF); and (d) cells differentiated in the presence of OF and treated daily with PEMF (PEMF + OF). The experimental setup was performed as indicated in Table 1. The SAOS-2 cell line was selected because it exhibits several fundamental osteoblast characteristics. Although, this cell line is derived from osteosarcoma and so could display some cellular behavior differences from primary human osteoblasts, it represents an accepted and representative model for in vitro osteogenic study [20], including studies of the multiple osteoblasts responses on new developed biomaterials [21,22].

### 3.1. Proliferation and Morphology of Osteoblast-Like Cells onto Wool Keratin Scaffold after PEMF Treatment

**Figure 1:**
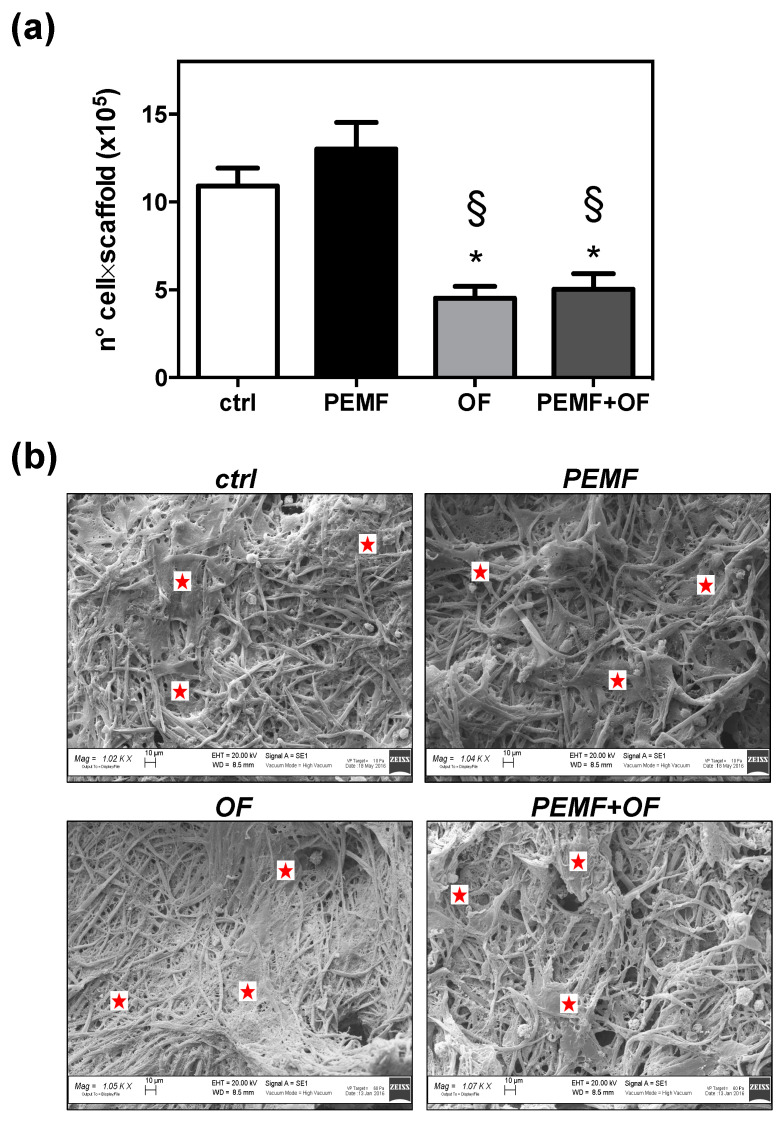
(**a**) Cell growth evaluation has been assessed by means of DNA quantification at day 21 of culture. Bars represent the mean values ± SD (standard deviation) of results (N = 3; n = 3, symbols indicate statistical significance vs. control (*) and vs. PEMF (§)). (**b**) Cell morphology assessed by SEM on all samples (scale bars = 10 μm; magnification: ctrl = 1020×; PEMF = 1040×; OF = 1050×; PEMF + OF = 1070×). Red stars indicate cell distribution on the wool keratin scaffolds in all conditions. In OF and PEMF + OF the dense layer of extracellular bone matrix (ECM) made difficult to discriminate a cell from another. Representative live cells visualized by fluorescein diacetate (FDA) staining on the scaffold’s surfaces are shown in Figure S3.

Firstly, the capability of the 3D fibrous structure of wool to support the cell adhesion and migration into the pore of wool keratin scaffold was confirmed by confocal laser scanning microscopy (Figure S1). Specifically, analysis of the orthogonal projections of CLSM images (Figure S1) displayed that osteoblasts were able to migrate into the pores of wool keratin scaffold after 24 h of incubation, that is a relevant event, since the migration and colonization into the scaffold may favor the bone matrix deposition. We selected PEMF with a frequency of 75 Hz and an intensity of 2 mT based on our previous study, demonstrating that these parameters improve the osteogenic differentiation of human mesenchymal stem cells (MSCs) [23]. The daily treatment with PEMF for 1 h was chosen as an exposure protocol on the basis of viability preliminary studies performed by using a single or daily PEMF dose (Figure S2). Next, cell proliferation on wool keratin scaffolds were assessed after 21 days of culture for the above treatments. The proliferation of SAOS-2 over 21 days was determined through a DNA quantification assay (Figure 1a). According to the data the addition of osteogenic factors, with or without the PEMF exposure resulted in a significant decrease of cell proliferation comparison both with ctrl and PEMF groups (* *p* < 0.05 and § *p* < 0.05, respectively). It can be seen that the proliferation of cells exposed to PEMF and OF simultaneously (PEMF + OF) showed comparable results with OF group (*p* > 0.05). Similarly, no significant difference was observed between ctrl and PEMF stimulated cells (*p* > 0.05). It is important to note that this may be an underestimation of the culture cellularity due to the trapping of DNA within the formed extracellular matrix and fibrous scaffold meshes. The SEM images show that the cells (indicated with red stars in the SEM images) adhered and spread onto the surface of the scaffolds after 21 days (Figure 1b), demonstrating that scaffolds are suitable frameworks for cellular attachment, spreading and proliferation and differentiation. All of these are crucial features for bone healing applications. However, differences were detected among the different experimental conditions. In comparison with ctrl and PEMF, due to OF and PEMF + OF stimuli, cells built their extracellular matrix (ECM) over the scaffold surface, which was tending to be hidden by a dense layer of cell-extracellular matrix (Figure 1b).

### 3.2. Bone Matrix Production by Human Osteoblast-Like Cells onto Wool Keratin Scaffold after PEMF Treatment

**Figure 2:**
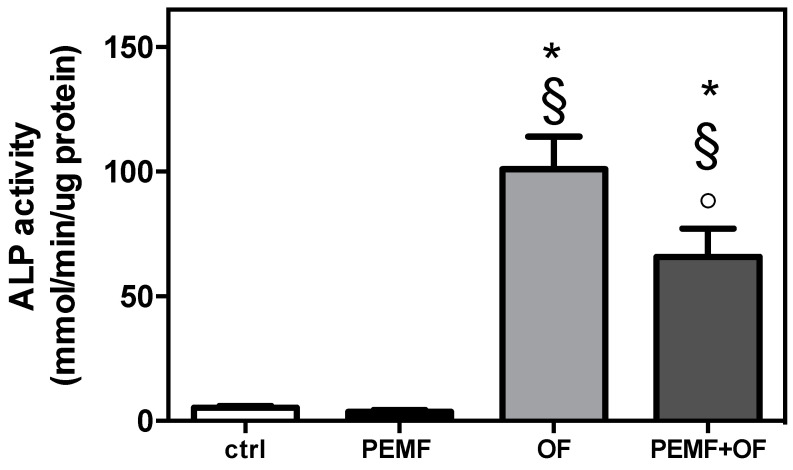
Alkaline phosphatase (ALP) activity after 21 days. ALP activity was colorimetrically determined, corrected for the protein content (measured with the BCA Protein Assay kit), and expressed as mM of p-nitrophenol produced per min per μg of protein. Bars express the mean ± SD (N = 3; n = 2, symbols indicate statistical significance vs. control (*), vs. PEMF (§) and vs. OF (°)).

To determine the action of daily PEMF exposure on human osteoblast-like cells cultured onto wool keratin scaffold, at the end of culture and treatment (21 days), all groups were assessed by evaluation of ALP activity, inorganic matrix production, bone-related protein gene expression and deposition. When the ALP activity was examined (Figure 2) it was found that both OF and PEMF + OF groups significantly increased the enzymatic activity over both ctrl and PEMF cultures (* *p* < 0.001 and § *p* < 0.001). The simultaneous treatment with PEMF and OF, however, significantly reduced the ALP activity when compared to the osteogenic factors’ sole exposure (° *p* < 0.001).

**Figure 3:**
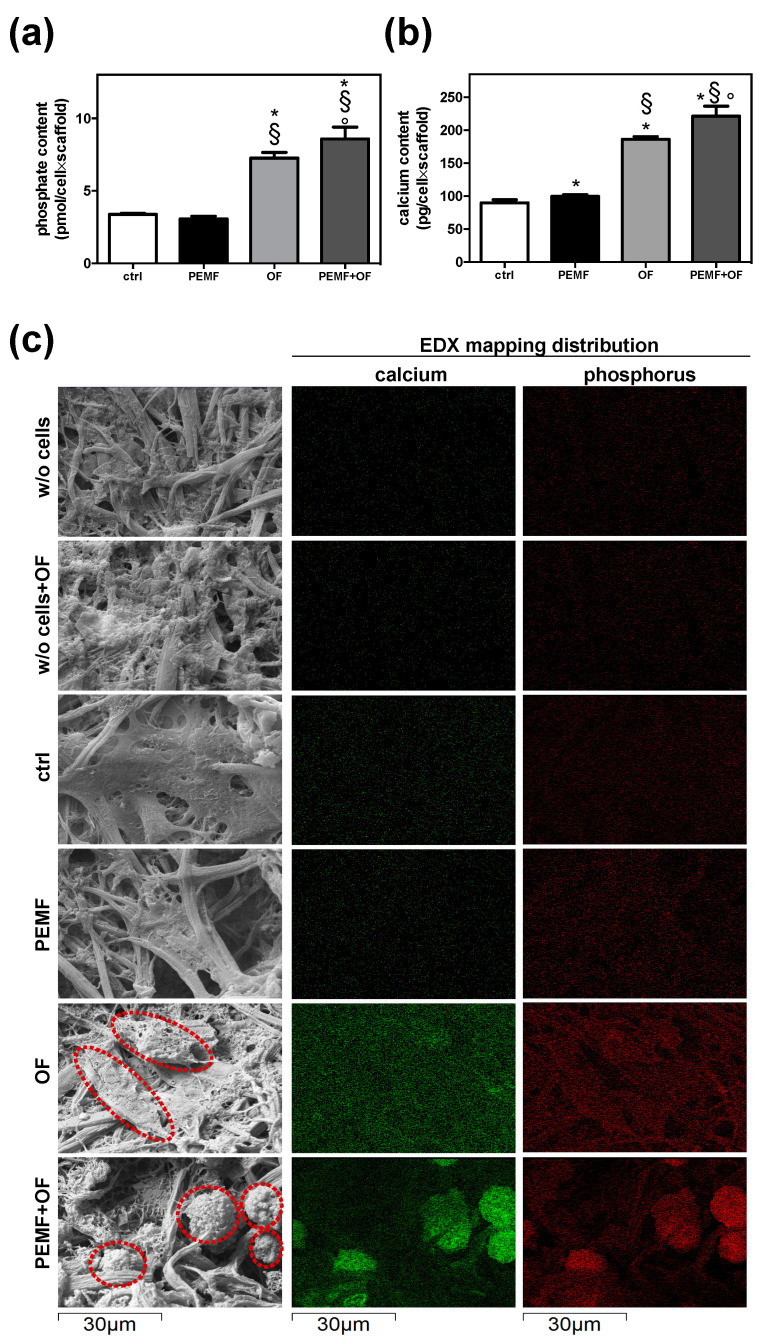
Quantification of inorganic matrix produced for 21 days. (**a**) Quantification of phosphate content by Phosphate Colorimetric Assay kit. Phosphate is measured in pmol/cell×scaffold; results are presented as mean ± SD (N = 3; n = 2; symbols indicate statistical significance vs. control (*), vs. PEMF (§) and vs. OF (°)). (**b**) Quantification of calcium content by the Ca^2+^/o-cresolphthalein complexone method. Results are expressed as pg/cell×scaffold and presented as mean ± SD (N = 3; n = 2, symbols indicate statistical significance vs. control (*), vs. PEMF (§) and vs. OF (°)). (**c**) Scanning Electron Microscopy with Energy Dispersive X-ray Spectroscopy (SEM-EDX) analysis of calcium and phosphorous deposits. Representative SEM pictures show the presence of inorganic deposits (indicated with red dotted circles) in particular in cells cultured in wool keratin scaffolds and treated for 21 days with PEMF + OF. EDX elemental mapping of calcium (green) and phosphorus (red) relative to the SEM pictures (dimension: 64.68 × 43.12 μm). All EDX analyses were conducted with an accelerating voltage of 20 kV and under low vacuum conditions. Wool keratin scaffolds cultured in maintenance medium in absence of cells (w/o cells) and with OF (w/o cells + OF) for 21 days were included as control. In both these conditions, the calcium and phosphorus signals were negligible.

Next, the quantification of the inorganic matrix produced was performed in all four conditions by the assessment of both phosphate and calcium content (Figure 3a,b). At day 21, the PEMF + OF group had the greatest phosphate content (ctrl, * *p* < 0.001; PEMF, § *p* < 0.001; OF, ° *p* < 0.01). Although no significant differences were detected between ctrl and PEMF samples (*p* > 0.05), a significant increase of phosphate was detected in PEMF + OF group and not in the OF group (° *p* < 0.01). In line with phosphate data, the analysis of calcium content reported that the calcium amount, expressed as pg of calcium per cell × scaffold, was greater in PEMF + OF samples than in the other conditions (* § ° *p* < 0.001). Additionally, the daily PEMF exposure significantly increased the calcium presence over ctrl (PEMF vs. ctrl, * *p* < 0.05) and over OF (PEMF + OF vs. OF, § *p* < 0.001). These quantitative results are corroborated by SEM-EDX elemental mapping (Figure 3c). Inorganic deposits were clearly identified by SEM imaging both in OF and PEMF + OF, but were absent both in ctrl and PEMF groups (Figure 3c). EDX elemental maps, in which calcium and phosphorus concentrations were mapped in green and red, respectively, revealed that the signal of both elements was remarkably higher and localized in specific region onto the wool keratin scaffold surfaces (indicated by the red dotted circles) treated with OF and PEMF + OF than the others (Figure 3c). In these latter conditions, both calcium and phosphorus signals were lower and appeared homogeneously distributed throughout the scaffold’s surface.

**Figure 4:**
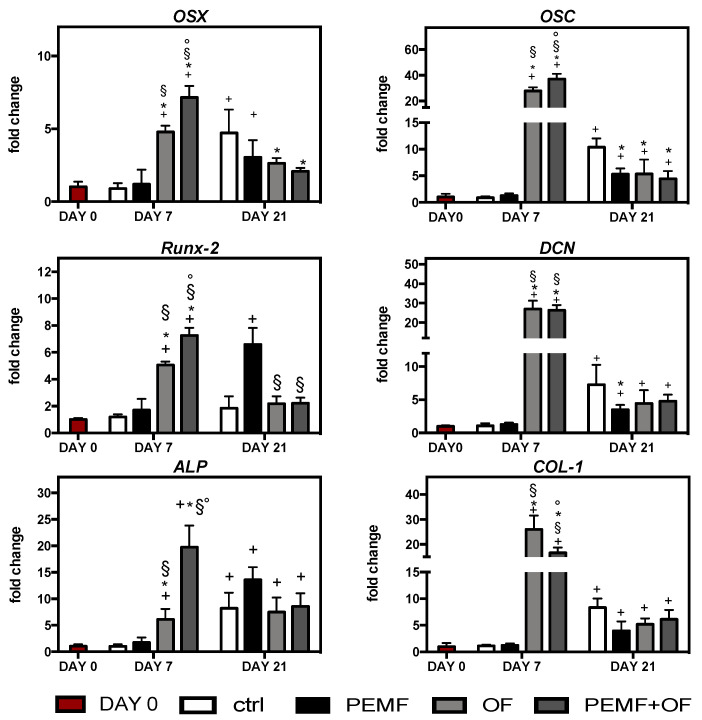
Gene expression of bone-specific markers as determined by quantitative reverse-transcription polymerase chain reaction (qRT-PCR) at day 7 and 21 of culture. Graphs show the fold change of gene expression relative to the expression in the cells at day 0. Symbols indicate statistical significance vs. day 0 (+), vs. control (*), vs. PEMF (§) and vs. OF (°) as determined by two-way ANOVA (N = 2, n = 3). Abbreviations: *OSX*, osterix; *Runx*-2, runt-related transcription factor-2; *ALP*, alkaline phosphatase; *OSC*, osteocalcin; *DCN*, decorin; *COL-I,* type-I collagen.

Subsequently, quantitative real-time PCR (qRT-PCR) analysis of the early and late osteogenic marker expression after 7 and 21 days was performed on the total RNA extracted from cells cultured in different conditions (Figure 4). In general, the addition of osteogenic factors in the maintenance media, with/without PEMF exposure, significantly enhanced osteogenic transcription factors levels in the cells cultured onto wool-keratin scaffold compared to day 0 (+ *p* < 0.05). This increase showed that variations of osteogenic genes were expression time dependent. During the early differentiation (day 1), the osteogenic gene expression was greatly upregulated in OF and PEMF + OF groups compared to ctrl and PEMF condition (* *p* < 0.05; § *p* < 0.05). Notably, expression of OSX, Runx-2, ALP and OSC gene expression was significantly increased by PEMF + OF compared with the OF group (° *p* < 0.05). At the same time, neither the control or PEMF group showed any stimulatory trend for osteogenic markers levels compared to day 0 (*p* > 0.05). Notably, at the end of the experimental procedure (21 days), expression of osteogenic genes declined in OF and PEMF + OF groups, but were still significantly higher than most genes tested on day 0 (+ *p* < 0.05), expect for Runx-2, which reached comparable levels of ctrl and day 0 (*p* > 0.05). Furthermore, on day 21 the stimulatory effect of PEMF in presence of OF was no longer apparent for the above-mentioned genes, with no significant difference between OF and PEM + OF condition (*p* > 0.05). It was interesting to observe that after 21 days of culture, the control group showed an increase of most analyzed genes (OSX, ALP, OSC, DCN, COL-1) in comparison with day 0 (+ *p* < 0.05). A similar trend was observed in PEMF exposure without osteogenic factors, with a strong upregulation of some genes related to the initial phases of osteogenic differentiation, like Runx-2 and ALP, compared with day 0 (+ *p* < 0.05). 

**Figure 5:**
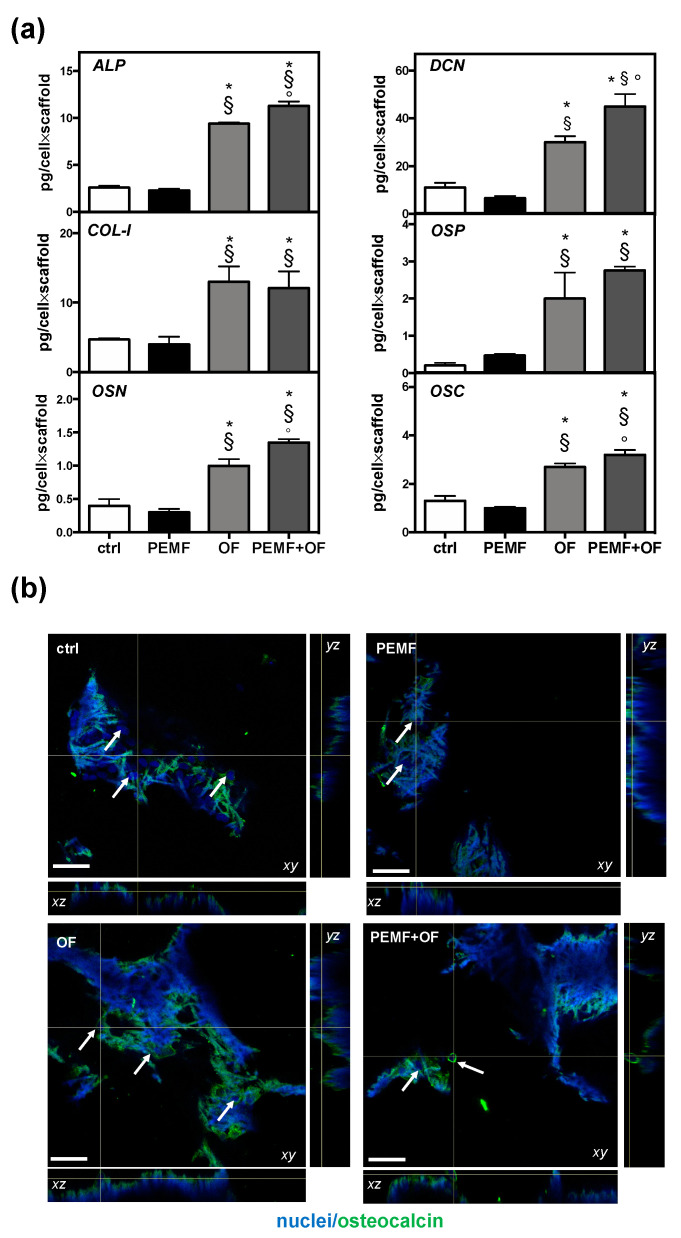
Bone osteogenic protein production by cells onto wool fibril sponges in the different conditions after 21 days of culture. (**a**) Quantification of indicated osteogenic proteins quantified by ELISA assay. Data are expressed as pg/cell×scaffold (N = 3; n = 2, symbols indicate statistical significance vs. control (*), vs. PEMF (§) and vs. OF (°)). (**b**) Representative orthogonal view of Confocal Laser Scanning Microscope (CLSM) images of bone osteocalcin immunolocalization (green) are shown with xy, yz, and xz planes. Nuclei (blue) were counterstained with Hoechst 33342. White arrows indicate the scaffold areas containing cells. Magnification 20×; the scale bar represents 50 µm. Negative control for non-specific staining of the secondary antibody and Tissue Culture Plates (TCPS) controls are shown in Figure S4.

Furthermore, the extracellular matrix constituents were extracted and quantified by ELISA assay at the end of the culture to characterize the osteogenic process. Remarkably, at the end of the study it emerged that the amount of bone extracellular matrix proteins produced by cells onto wool fibril sponges resulted significantly higher in osteogenic groups (with or without PEMF) than in the control and PEMF-treated cells (* *p* < 0.05 and § *p* < 0.05, Figure 5a). Furthermore, the highest production was detected in cells exposed daily to PEMF with osteogenic factors added in the culture medium (PEMF + OF) over all the other samples. Noteworthy, compared to OF, a significant enhancement of ALP, OSN, DCN, and OSC deposition was found in PEMF + OF samples (° *p* < 0.001, ° *p* < 0.01, ° *p* < 0.01, ° *p* < 0.05, respectively). By contrast, no significant difference was measured between the control and PEMF-treated cells for all bone proteins measured (*p* > 0.05). Lastly, although the thick and intricated structure of the 3D wool keratin scaffolds presented challenges, the orthogonal projections of CLSM images confirmed the presence of cell (nuclei in blue), showing that they were able to migrate and infiltrate into the full depth of the scaffold and colonize its porosities. Agreeing with ELISA data, immunofluorescence images showed a clear and marked osteocalcin fluorescence signal, in particular in cells differentiated in OF and PEMF + OF treatments (Figure 5b).

### 3.3. Mechanical Properties of Wool Keratin Scaffolds after Cell Growth/Differentiation and PEMF Treatment

**Figure 6:**
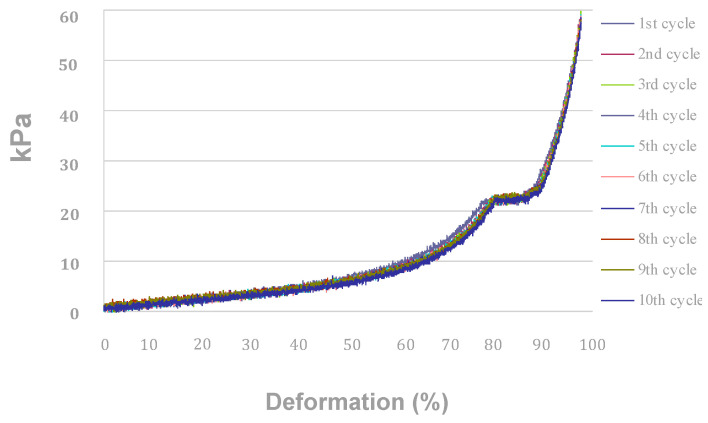
Compression traces of the wool fibril sponges in the wet state after 21 days of culture in MM with cells and PEMF. Similar behavior was obtained for all other conditions tested.

During compression cycles carried out in the dry state, wool keratin scaffolds (or sponges) showed a permanent deformation not evident in sponges compressed in wet conditions [4]. Since their application is forecast to be performed in wet conditions (inside body), the compression behavior was evaluated in wet conditions after 21 days of immersion in a culture medium. The compression behavior of sponges submitted to 10 repeated compression cycles in a wet state, after 21 days of immersion in MM with or without cells or PEMF, has been evaluated (Figure 6, Table 2).

After 21 days of culture in maintenance (MM) and osteogenic (OF) medium a resilient behavior was still observed: compression traces are almost overlapping each other, and no permanent deformation can be detected (Figure 6). All compression traces display a horizontal line in the load range 22–24 kPa, which is most likely due to reversible crushing deformation of the pore structure of sponges (Figure 6). In other words, water filled the macro- and micro-pores and penetrated into the amorphous keratin domains as well, resulting in increased elasticity of the whole structure. Nevertheless, for compression loads higher than 24 kPa, the structure of the wool fiber assembly would not represent a sponge anymore. Thus, the compression moduli of sponges have been compared in the load ranges 2–8 kPa and 10–22 kPa (Table 2). 

### 3.4. Ageing Test

**Figure 7:**
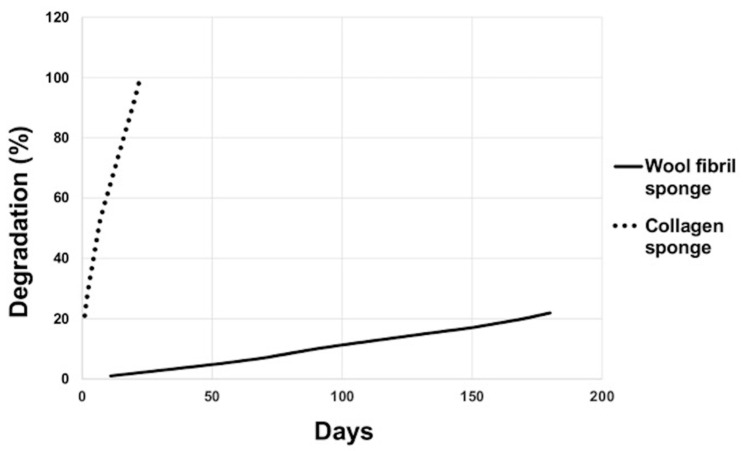
Degradation rate of the commercial collagen sponge vs. wool fibril sponge.

Both wool keratin scaffolds and commercial collagen sponges (reference polymeric material) were immersed into the same Ringer’s solution (ageing reference solution for bone biomaterials) at 37 °C. The degradation rate of collagen sponges was significantly higher when compared with the degradation rate of the wool scaffolds. The collagen sponges degraded completely in 22 days, whereas the wool sponges exhibited a degradation rate of only 22% only after 180 days (Figure 7).

## 4. Discussion

Our study investigated the effects of electromagnetic stimulation on osteoblasts seeded onto wool keratin scaffolds in terms of growth, differentiation, and bone extracellular matrix production.

Most of the other materials used as scaffolds for the repair of bone defects offer only a feasible structure within which to heal or regenerate and do not induce or boost the regeneration processes. Among the biomaterials, bioceramic scaffolds, mainly from calcium phosphates, are widely employed for bone regeneration because of their chemical similarity to the bony inorganic matrix which confers osteoconductive properties, increasing scaffold integration inside tissue and, as a consequence, leading to the bone healing. A large number of strategies have and are been investigated in order to improve the interaction of biomaterial with bone tissue. The use of PEMF might be a potential adjuvant treatment to enhance the osseointegration process. The effects of PEMF on osteoblasts onto different biomaterials have been investigated in several in vitro and in vivo studies [14]. PEMFs act on osteoblasts, improving their growth, differentiation and expression of a mature phenotype on implanted materials and, as consequence, promote tissue integration [24,25,26]. Most of these researches were focused on titanium and titanium alloy-based biomaterials [7,23,25,27,28,29,30], but similar effects were also achieved after PEMF application on cells cultured onto bioceramic and polymers scaffolds [31,32,33].

The electromagnetic stimulus of this study was demonstrated [8,9] to elicit time varying mechanical forces acting perpendicularly or tangentially onto the cell membrane. For instance, osteoblasts are susceptible to fluid shear stress and react with an enhanced transcription of bone matrix genes [11,12]. Traction and compression vary the activities of intracellular signaling molecules such as Rho GTPases, guanine nucleotide exchange factors, GTPase activating proteins, and the MAPK pathway, consequently modulating the expression of transcription factors essential for the homeostasis of bone [13]. In addition, several studies demonstrated that the electromagnetic fields are accompanied by increases in cytosolic calcium concentration and might involve calcium/calmodulin pathway [34]. According to Pavalko’s diffusion-controlled/solid-state signaling model, the increase of the cytosolic calcium concentration is a starting point of signaling pathways targeting specific genes of the bone matrix [11].

In agreement with this model, our previous findings on human mesenchymal stem cells (hMSCs) showed that the daily PEMF exposure affected cells osteogenesis by interfering with selective calcium-related osteogenic pathways [35], including when they were seeded on TiO_2_ nanostructures surfaces [23]. In line with these studies, our previous findings on human mesenchymal stem cells (hMSCs) showed that the daily PEMF exposure affected cells osteogenesis by interfering with selective calcium-related osteogenic pathways [35], including when they were seeded on TiO_2_ nanostructures surfaces [23].

We note, however, that though the effects of PEMF stimuli on cells are well documented, the mechanism of action is still unclear. The broad range of settings used for PEMFs stimulation represents an obstacle, making it difficult to compare the results in the literature. It has been demonstrated, for example, that the time of PEMF exposure could be a critical parameter [36]. We previously observed that exposure to PEMF for 10 min per day at the same time increased, on hMSCs, the extracellular matrix secretion as well as enhancing the osteogenic gene expression [23]. Another study on a rat model found that 1 h of exposure significantly improved the bone healing process compared with longer treatment [37]. Moreover, other variables can affect PEMF outcomes, including the types, density, and differentiation stage of cells [38] as well as the medium composition (e.g., serum percentage and osteogenic supplements) [39]. For these reasons, there are contradictory results. PEMF can directly stimulate osteoprogenitor cells towards osteogenic differentiation at the expense of proliferation [40], or enhance both hMSCs osteogenic differentiation and proliferation [41]. Besides, Martino et al. reported that repetitive PEMF exposure increased the SAOS-2 matrix mineralization without affecting cell proliferation [42]. In agreement with Martino et al., in our experimental settings, we observed that, the daily application of pulsed electromagnetic field slightly affected SAOS-2 growth (both with or without osteogenic factors), but that osteogenic differentiation and mineralization significantly increased only in combination with osteogenic agents. The number of proliferated cells and their differentiation should be important parameters to estimate the biological effect of PEMF on cells onto the wool keratin scaffold. According to our results, cell proliferation increased with culture time in all conditions tested (ctrl, PEMF, OF, and PEMF + OF), demonstrating that the scaffold has good biocompatibility. However, at the end of culture, the proliferation resulted lower in the groups cultured in the presence of the osteogenic factors, with or without PEMF, suggesting a link with the osteogenic factors’ addition. Commonly, in vitro osteogenic factors (e.g., dexamethasone and β-glycerophosphate) are directly introduced into the culture medium to drive the cells toward osteogenic differentiation: dexamethasone is a synthetic glucocorticoid that activates the osteogenic lineage differentiation and β-glycerophosphate acts as a source of phosphate with an important role in the mineralization process. At the beginning of the osteogenic differentiation, cells undergo a proliferation process that stops when the differentiation starts [43]. Additionally, this phenomenon may clarify the small increase of proliferation found in OF and PEMF + OF groups at the end of the experiments.

Again, because of variables mentioned, the effects of PEMF described in the literature vary considerably, with significant variations in the determination of the peak. Tsai et al. observed an increase of ALP activity on hMSCs after PEMF stimulation on day 7, but not on day 3 and 10 [44], whereas Jansen et al. reported that ALP activity was not significantly affected by PEMF treatment and the peak significantly varied between bone marrow mesenchymal stem cells from different donors [40]. After 21 days, we detected a lower ALP activity in PEMF + OF cultures than the OF samples. During osteogenic differentiation, in the early stages, there is an initial peak in ALP activity, which is followed by a subsequent reduction as the cells mature and lay down mineral [45,46]. Here, it is possible to speculate that the decrease of ALP activity observed in PEMF + OF group over OF may be correlated with the fact that, after 21 days, the cells were in the late stage of differentiation as a consequence of both osteogenic factors and PEMF stimulation, well-known to accelerate and boost the osteogenic differentiation and mineralization [47]. It is probable that we overlooked and missed the PEMF induced stimulation of ALP activity, which could have occurred earlier than 21 days.

The ECM deposition and calcification represent optimal indicators for assessing the in vitro maturation of osteoblastic phenotype [48]. Notably, in our in vitro system, the presence of the osteogenic factors seemed to be critical for the osteogenic commitment in PEMF-treated groups. SAOS-2 cells stimulated by PEMF, without osteogenic factors, clearly displayed comparable levels of mineralization with ctrl groups. By contrast, when cultured in medium containing osteogenic ingredients, PEMF-treated cells increased both phosphate and calcium deposition. SEM images and EDS mapping of the elements Ca, P, and Mg corroborated the idea that the new bone matrix was ultimately deposited onto the surface of the wool keratin scaffold. Also, the osteogenic factors determined an enhancement of both calcium and phosphate contents, but the OF-groups merely presented a homogeneous distribution of both elements onto the scaffold surface. The formation of mineral nodules, mainly in PEMF + OF cases, might be ascribable to the accelerated differentiation exerted by the synergic action of PEMF and OF, a point which was also confirmed by the quantification of bone-related proteins. We observed that PEMF exposure significantly increased the production of some proteins related to the matrix deposition and mineralization (e.g., ALP, OSN, DCN, and OSC), preferentially in osteogenic medium (PEMF + OF group). The enhancement of the proteins implicated in bone formation and remodeling further supports the hypothesis that PEMF, in combination with osteogenic signals, promotes the production of proteins necessary for mineralization (ALP makes the phosphates available for calcification, OSN is a calcium- and collagen-binding ECM glycoprotein acting as a modulator of cell-matrix interactions, DCN orchestrates the correct collagen fibril assembly, OSC is the most specific marker for osteoblasts maturation [49]).

The qRT-PCR analysis appears to confirm the speeding-up effects towards a fully osteoblastic mature state exerted by the simultaneous presence of OF and PEMF. Indeed, during the early differentiation (day 7), we determined an up-regulation of osteogenic markers in both OF and PEMF-treated groups. At day 21 of culture, we found a decrease of the expression of both early (Runx-2, OSX, ALP) and late bone-related genes (COL-I, DCN, OSC) in particular in both PEMF + OF and OF condition than control and PEMF group. These findings strongly suggest that the PEMF + OF exposition seems to accelerate the cells toward the bone differentiation pathway influencing all the proteins involved in terms of transcription and translation in a significant manner. Hypothetically, the bone protein transcription was immediately activated after the osteogenic addition, with or without PEMF exposition, which allowed protein translation. Perhaps the fast reaching of a mature phenotype from the cells in these conditions forced them to downregulate and prematurely stop the protein transcription with respect to protein translation. Interestingly, in the ctrl and PEMF groups, cells could be in an early stage of differentiation, and so result in a higher level of bone gene expression over OF and PEMF + OF, that may be due to spontaneous differentiation of the cells after 21 days of culture in response to the porous topography of the scaffold. In general, similarly to a previous study where PEMF alone did not affect cell mineralization or cell viability in osteoblast-precursor MC3T3-E1 cells [50], we found that SAOS-2 cells matrix mineralization was heavily influenced by the PEMF exposure only when the medium was supplemented with the osteogenic factors. Furthermore, keratin presents peculiar tri-peptide motives relevant for cell binding, such as leucine-aspartic acid-valine (LDV), glutamic acid-aspartic acid-serine (EDS), and arginine-glycine-aspartic acid (RGD). Indeed, these motives are found in several extracellular matrix proteins, like fibronectin [51] and their interaction with cells can elicit different responses [52]. These responses might overlap with the signaling regulation exerted by PEMF and/or osteogenic factors. As reported by Klontzas et al. [53], alginate hydrogels crosslinked with a specific tri-peptide (glycine-histidine-lysine or GHK, characterizing the bone ECM protein osteonectin) significantly induced osteogenic differentiation because of the presence of this GHK peptide. Hence, we can speculate the different mechanical/biochemical stimuli utilized in the present study are acting on the very same or closely interconnected metabolic pathways. To corroborate or refute this hypothesis, further studies should focus on the specific action the individual stimulus has on the osteogenic differentiation pathways. Besides, precise and analytic approaches, such as mass spectrometry-based metabolomics and proteomics could be properly employed for these purposes.

In summary, we have demonstrated the importance of including both physical and biochemical factors for fostering the osteogenic differentiation. PEMF (frequency of 75 Hz, magnetic induction of 2 mT, 1 h of exposure per day) stimulated in the osteogenic cultures the highest production of bone extracellular matrix and, therefore, it could be very useful in combination with osteogenic factors during the cell culture onto wool keratin scaffolds. Finally, no significant differences were detected in mechanical behavior between scaffolds with or without cells, in maintenance or osteogenic medium and with or without PEMF. The presence of SAOS-2 cells influenced neither the compression behavior nor the resilience of keratin scaffolds in wet conditions. As expected, the wool fibril sponges showed high chemical and physical stability after 21 days of culture, despite the fact that they consist of proteins (biodegradable polymers). Ageing tests revealed that wool fibril sponges, characterized by an exceptional number of crosslinks stabilizing the keratin structure are very stable and show a lower degradation rate than commercial collagen sponges. This suggests that these materials have a promising application for long-term support of in vivo bone formation (e.g., as biocompatible, bone-proteins rich, bone-cells rich fillers for bone defects). In the future, experiments involving a higher number of scaffolds may help to study more precisely the not significant results observed in our study.

## 5. Conclusions

With the awareness that in vivo studies will confirm the feasibility and the bone healing potentiality of the integrated bioengineering approach proposed in this paper, we can conclude by stating that this is the first in vitro evidence of the positive advantages of this approach: the capability of the PEMF to boost the osteogenic differentiation in synergy with osteogenic factors made the 3D wool keratin an osteoconductive biomaterial, which, once tested in in vivo models, may elicit positive bone response and may speed up tissue healing after surgery. Prospectively, this experimental design may be employed to electromagnetically stimulate the differentiation of bone marrow mesenchymal stem cells toward the osteogenic phenotype onto keratin substrates, subsequently leading to a potential application in regenerative medicine.

## Figures and Tables

**Table 1:** Experimental set up to investigate the effect of the daily pulsed electromagnetic field (PEMF) treatment on human SAOS-2 osteoblast-like cells seeded onto porous wool keratin scaffold for 21 days.

Experimental Condition	PEMF * Exposure Protocol	In Vitro Investigation
**Ctrl** (maintenance medium, MM)	not exposure °	**Day 7:** qRT-PCR bone gene expression **Day 21:** DNA content SEM analysis ALP activity Phosphate content Calcium content Bone proteins: qRT-PCR and ELISA Confocal Laser Scanning Analysis Mechanical characterization
**PEMF** (MM + PEMF)	1 h per day up to 21 days °	
**OF** (MM + OF)	not exposure °	
**PEMF + OF** (MM + OF + PEMF)	1 h per day up to 21 days °	

* The following parameters were adopted: magnetic induction amplitude of 2 ± 0.2 mT, frequency of 75 ± 2 Hz, pulse duration of 1.3 ms. The electromagnetic bioreactor was placed into a standard cell culture incubator in a 37 °C, 5% CO_2_ environment. ° The medium was changed every 3 days.

**Table 2:** Mechanical characterization of wool fibril sponges in the wet state after 21 days in different culture conditions.

Experimental Condition	Compression Range (kPa)	Average Modulus (kPa)
without cells in MM	2–8	18.0 ± 5.2
	10–22	11.1 ± 8.3
without cells in PEMF	2–8	14.1 ± 2.2
	10–22	85.2 ± 8.1
without cells in OF	2–8	21.3 ± 3.1
	10–22	80.4 ± 7.4
without cells in PEMF + OF	2–8	22.1 ± 3.1
	10–22	54.4 ± 4.6
with cells in MM (ctrl)	2–8	24.2 ± 2.2
	10–22	102.6 ± 9.1
with cells in PEMF	2–8	16.2 ± 2.7
	10–22	58.3 ± 9.2
with cells in OF	2–8	27.4 ± 30.1
	10–22	90.5 ± 90.2
with cells in PEMF + OF	2–8	25.1± 3.3
	10–22	59.3± 8.1

## Supplementary Materials

The following are available online at https://www.mdpi.com/1996-1944/13/14/3052/s1, Figure S1: CLSM morphological analysis of osteoblast-like cells seeded onto the wool fibril sponges, Figure S2: Viability of SAOS-2 cells cultured onto the wool keratin scaffolds and exposed to different PEMF doses, Figure S3: Representative CLSM images of live cells onto wool fibril sponge in the different experimental conditions, Figure S4: Representative CLSM images of negative control for non-specific staining of the secondary antibody and TCPS controls, Table S1: Primers used for qRT-PCR study.
