# Supplementary material for: In Vitro Production of Calcified Bone Matrix onto Wool Keratin Scaffolds via Osteogenic Factors and Electromagnetic Stimulus
Source: Materials (Basel). 2020 Jul 8;13(14):3052. doi: 10.3390/ma13143052 (PMC7411850; doi:10.3390/ma13143052)
Supplement: Supplementary file 1 [file materials-13-03052-s001.pdf]

materials

Supplementary Materials: In Vitro Production of Calcified Bone Matrix onto Wool Keratin Scaffolds via Osteogenic Factors and Electromagnetic Stimulus

Nora Bloise [1,2,*], Alessia Patrucco [3], Giovanna Bruni [4], Giulia Montagna [1,5], Rosalinda Caringella [3], Lorenzo Fassina [5], Claudio Tonin [3] and Livia Visai [1,2,*]

1 Department of Molecular Medicine (DMM), Centre for Health Technologies (CHT), UdR INSTM, University of Pavia, Viale Taramelli 3/B-27100 Pavia, Italy; giulia.montagna04@universitadipavia.it
2 Department of Occupational Medicine, Toxicology and Environmental Risks, Istituti Clinici Scientifici (ICS) Maugeri, IRCCS, Via Boezio 28-27100 Pavia, Italy
3 Institute of Intelligent Industrial Technologies and Systems for Advanced Manufacturing (STIIMA), Italian National Research Council (CNR), Corso Pella 16-13900 Biella, Italy; a.patrucco@stiima.cnr.it (A.P.); linda.car87@yahoo.it (R.C.), c.tonin@stiima.cnr.it (C.T.)
4 Center for Colloid and Surface Science (C.S.G.I.), Department of Chemistry, Section of Physical Chemistry, University of Pavia, Viale Taramelli 16-27100 Pavia, Italy; giovanna.bruni@unipv.it
5 Department of Electrical, Computer and Biomedical Engineering (DIII), Centre for Health Technologies (CHT), University of Pavia, Via Ferrata 5-27100 Pavia, Italy; lorenzo.fassina@unipv.it
* Correspondence: nora.bloise@unipv.it (N.B.); livia.visai@unipv.it (L.V.); Tel.: +39-0382-987725 (L.V.)

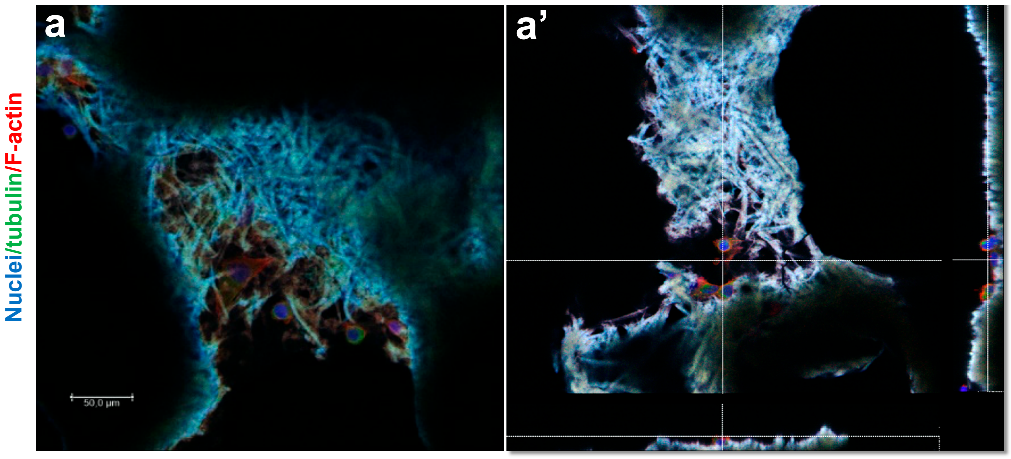

Figure S1. CLSM morphological analysis of osteoblast-like cells seeded onto wool fibril sponges. (**a**) After 24 h from seeding, the cytoskeleton organization was observed by F-actin staining with Phalloidin-TRITC (in red) and α-tubulin (in green). Nuclei were stained with Hoechst 33342 (in blue). Orthogonal view of CLSM image is shown in panel (**a’**) Scale bar = 50.0 µm.

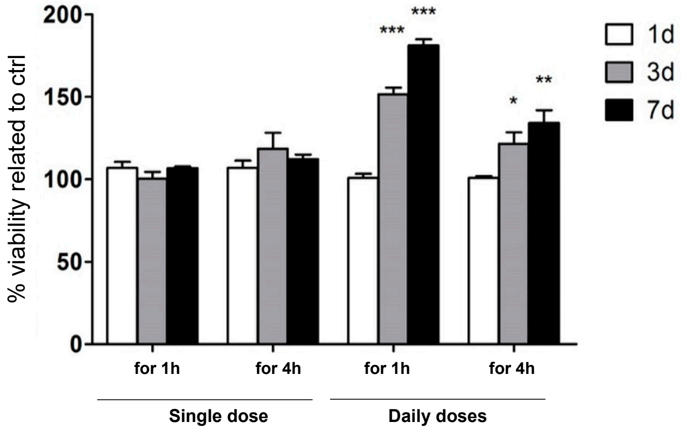

Figure S2. Viability of SAOS-2 cells cultured on wool keratin scaffolds and exposed to different PEMF doses. Cell viability is expressed as percentage of cell viability assessed after the different PEMF applications related to control (untreated). Results are presented as mean ± SD (* $p < 0.05$; ** $p < 0.01$; *** $p < 0.001$).

The stimulation protocol was selected on the basis of previous results [1–3] and of preliminary evaluation performed stimulating cells with PEMF for 5, 10, and 30 min per 1 day (single dose) or per 1 day for 7 days (daily doses) in maintenance media. Viability was assessed after 1, 3 and 7 days. As no effects were observed in terms of cell viability in comparison with the untreated control, PEMF stimulation was performed increasing the time of stimulation, testing a single or daily dose (Figure S2). PEMF exposure for 1h per day was selected because it determined a significant increase of cell viability over time, which proved the activation of cell response to PEMF stimulus, which is a crucial event for subsequent osteogenic differentiation studies.

The parameters were adopted: magnetic field 2 ± 0.2 mT, induced electronic tension amplitude 5 ± 1 mV, frequency of 75 ± 2 Hz, pulse duration 1.3 ms. In clinical settings the PEMF parameters were similar, but a period of 30–40 min represented the exposure time for tissues or organs.

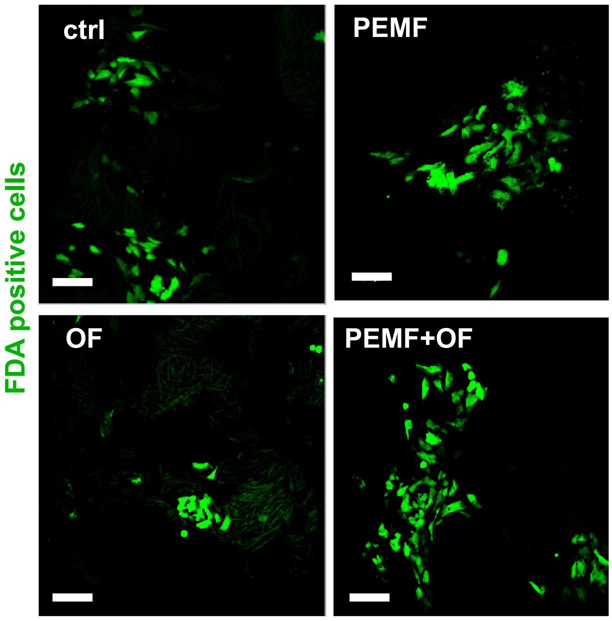

Figure S3. Representative CLSM images of live cells onto wool fibril sponge in the different experimental conditions. Live cells were visualized with FDA staining as described in Materials and Methods section (40× magnification, scale bar = 50 μm).

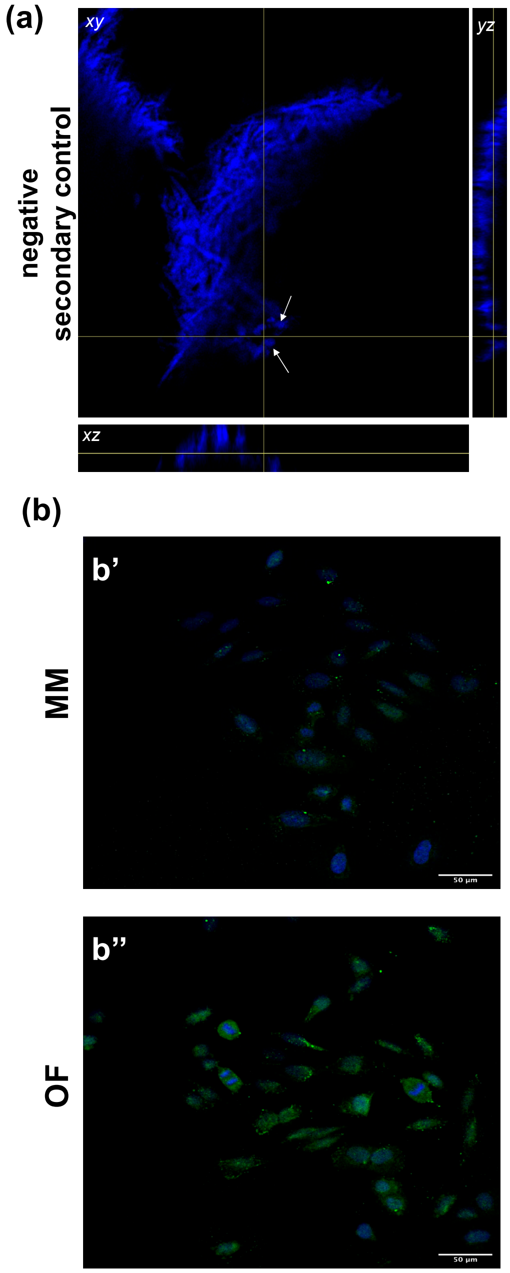

Figure S4. Representative CLSM images of negative control for non-specific staining of the secondary antibody (**a**) and TCPS controls (**b**). (**a**) SAOS-2 cultured onto wool fibril sponge, incubated overnight at 4 °C with PAT instead of the anti-osteocalcin primary antibodies, and with Alexa Fluor 488 goat anti-rabbit IgG (Molecular Probes). Representative orthogonal view of CLSM images are shown with xy, yz, and xz planes. Nuclei (blue, indicated with white arrows) were counterstained with Hoechst 33342 (2 µg/mL). Magnification 20×. (**b**) CLSM images of bone osteocalcin immunolocalization on TCPS, after 21 days in MM (**b′**) and with osteogenic factors (**b″**). Magnification 40×; the scale bar represents 50 µm.

Table S1. Primers used for qRT-PCR study. GAPDH was the housekeeping gene.

Genes	Upstream Primer Forward 5′-3′	Downstream Primer Reverse 5′-3′	Amplicon Size (bp)
**ALP**	CTA TCC TGG CTC CGT GTC C	AGC CCA GAG ATG CAA TCG	138
**COL-I**	CAT GTT CAG CTT TGT GGA CC	TTC TGT ACG CAG GTG ATT GG	128
**DCN**	CGA GTG GTC CAG TGT TCT GA	AAA GCC CCA TTT TCA ATT CC	400
**GAPDH**	AGC CTC AAG ATC ATC AGC AAT GCC	TGT GGT CAT GAG TCC TTC CAC GAT	120
**Runx-2**	ACA GTA GAT GGA CCT CGG GA	ATA CTG GGA TGA GGA ATG CG	113
**OSC**	AAG AGA CCC AGG CGC TAC CT	AAC TCG TCA CAG TCC GGA TTG	107
**OSX**	CTC AGC TCT CTC CAT CTG CC	GGG ACT GGA GCC ATA GTG AA	99

References

1. Fassina, L.; Visai, L.; Benazzo, F.; Benedetti, L.; Calligaro, A.; Cusella De Angelis, M.G.; Farina, A.; Maliardi, V.; Magenes, G. Effects of electromagnetic stimulation on calcified matrix production by SAOS-2 cells over a polyurethane porous scaffold. *Tissue. Eng.* **2006**, *12*, 1985–1999.
2. Bloise, N.; Petecchia, L.; Ceccarelli, G.; Fassina, L.; Usai, C.; Bertoglio, F.; Balli, M.; Vassalli, M.; Cusella De Angelis, M.G.; Gavazzo, P.; Imbriani, M.; Visai, L. The effect of pulsed electromagnetic field exposure on osteoinduction of human mesenchymal stem cells cultured on nano-TiO_2 surfaces. *PLoS. One.* **2018**, *13*, e0199046.
3. Ceccarelli, G.; Bloise, N.; Mantelli, M.; Gastaldi, G.; Fassina, L.; Cusella De Angelis, M.G.; Ferrari, D.; Imbriani, M.; Visai, L. A comparative analysis of the *in vitro* effects of pulsed electromagnetic field treatment on osteogenic differentiation of two different mesenchymal cell lineages. *Biores. Open Access* **2013**, *2*, 283–294.

© 2020 by the authors. Licensee MDPI, Basel, Switzerland. This article is an open access article distributed under the terms and conditions of the Creative Commons Attribution (CC BY) license (http://creativecommons.org/licenses/by/4.0/).
